# Exploratory Analysis of the Sasang Constitution by Combining Network Analysis and Information Entropy

**DOI:** 10.3390/healthcare10112248

**Published:** 2022-11-10

**Authors:** Won-Yung Lee, Sang Hyuk Kim, Siwoo Lee, Young Woo Kim, Ji-Hwan Kim

**Affiliations:** 1 School of Korean Medicine, Dongguk University, 32 Dongguk-ro, Ilsandong-gu, Goyang-si 10326, Korea; 2 Korean Medicine Data Division, Korean Institute of Oriental Medicine, Yuseong-daero, Daejeon 34054, Korea; 3 School of Korean Medicine, Gachon University, Seongnam 13120, Korea

**Keywords:** Sasang constitutional medicine, network-based analytics, entropy, personalized medicine

## Abstract

Sasang constitutional medicine is a unique concept in Korean medicine that can provide valuable insights into personalized healthcare and disease treatment. In this study, we combined network analysis and information entropy to systematically investigate the related information of Sasang constitutional (SC) types. A feature network was constructed using SC type and clinical information. The SC type-associated features and feature classes were identified using statistical analysis and entropy ranking. The patient network was constructed based on SC-type-associated features. We found that the feature network was closely connected within the features of the same classes and between several feature class pairs, including the symptom class. Most of the separation values between the feature classes, including the symptom class, were negative. In addition, we found 42 clinical features related to the SC type, and two important classes -personality and cold/heat- that increase the entropy ranking of the SC type. In the patient network, we found sparsely connected modules between SC types and a positive separation value between the Taeeumin–Soeumin and Taeeumin–Soyangin pairs. Our data-driven approach provides a deeper understanding of modernized forms of SC types and suggests that SC type is a practically useful concept for stratified healthcare and personalized medicine.

## 1. Introduction

The growing recognition of personalized medicine by clinicians, the pharmaceutical industry, and patients highlights the need for tailored approaches for individual patients [1]. Personalized medicine aims to provide customized medical treatments for individual patients by integrating information from diverse data sources such as individual phenotypes, lifestyles, and clinical information. Sasang constitutional medicine (SCM) is a personalized form of Korean medicine (KM). SCM experts diagnose a patient’s constitution by considering the appearance, personality, symptoms, and other characteristics of the individual. Its constitution is called the Sasang constitution (SC) type and consists of four types: Taeeumin (TE), Soeumin (SE), Soyangin (SY), and Taeyangin (TY). To modernization of SCM, researchers have developed tools for the diagnosis of SC type and investigated the relationship between SC type and disease (See Related works). The core ideas of SCM share much with the aim of personalized medicine and can provide valuable insights and experiences that can be helpful for the improvement of personalized medicine [2,3]. However, since SCM theory is composed of its own language and form, it is difficult for other healthcare providers to accurately understand the concept of SC type.

**Figure 1:**
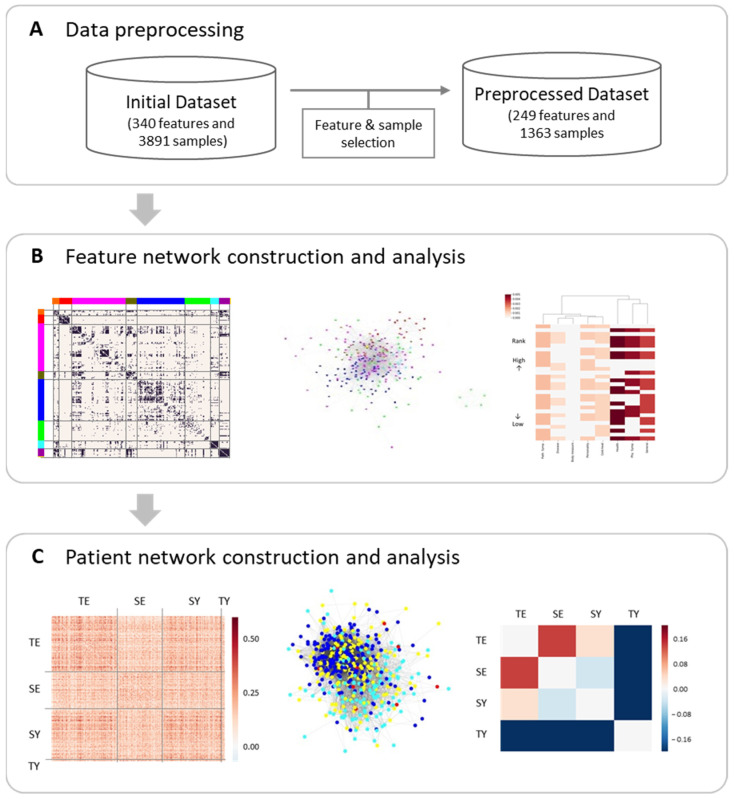
Overall research strategy of the network analytical approach: (**A**) data preprocessing was conducted by selecting features with high recording rates and associations to the SC type (see methods). The feature network (**B**) and patient network (**C**) were then constructed and analyzed, in which the nodes were defined as clinical features or the subjects, and the edges between nodes were computed using adjusted MIs between features or subjects.

In this study, we conducted an exploratory analysis to capture insights into the SC type (Figure 1). The highlights of our study to address the above-mentioned problem can be summarized as follows. We tried to comprehensively and quantitatively analyze how modern SCM experts define the SC types and the relevance of each SC type. To this end, we combined network analysis and information entropy. Those approaches have been applied to understand complex systems, such as social networks and biological phenomena. Network construction and analysis of high-dimensional data can provide higher-level information such as network topology, which cannot be identified by conventional analysis [4,5,6,7]. In addition, information entropy can be a quantitative measure of uncertainty regarding an outcome derived from a probability distribution [8,9]. We believe that our study can propose the concept of the SC types that is easy to understand even for healthcare providers in other fields.

This study was performed using a multicenter study dataset collected by the Korean Institute of Oriental Medicine. First, we constructed and analyzed a feature network consisting of SC type and KM clinical information such as general health, physiological status, body type, and disease. Second, we explored the features and feature classes primarily related to SC types using statistical analysis and entropy ranking, respectively. Finally, we constructed and investigated patient networks based on the similarity of SC-associated features between men and women.

The rest of this paper is organized as follows. Section 2 describes the related works on investigating clinical information associated with SC types and methods of investigating clinical information. Section 3 presents the dataset and analysis method used in this study. Section 4 demonstrates the results and discussion is illustrated in Section 5.

## 2. Related Works

Researchers have identified associated factors related to the Sasang constitution or developed tools to diagnose SC types using clinical data. For instance, the results of a multicenter survey suggested that the SC type may be a risk factor for certain chronic diseases, such as diabetes, abdominal obesity, and prehypertension [10,11,12,13]. Another cohort study evaluated dietary behaviors according to SC type [14]. In addition, a clinical study of hospital patients investigated differences in urinary incontinence function according to SC type and cold-heat subgroup [15]. On the other hand, researchers have developed diagnostic tools for SC types such as SCAT or K-prism using the patient’s body type, questionnaire, and voice information [16,17,18,19].

However, as shown in Table 1, most studies have focused on investigating associated factors between a small set of predefined features under specific hypotheses, limiting the possibility of generating new hypotheses from multidimensional data. Park et al. used a machine learning approach to investigate key features by SC type, but this mainly focused on uncovering factors that contributed to the diagnostic procedure. Therefore, there is still room for investigating the related information for SC type in a hypothesis-free manner.

## 3. Materials and Methods

### 3.1. Data Collection and Feature Selection

The dataset was obtained from the Korean Medicine Data Center (KDC) of the Korea Institute of Oriental Medicine. The KDC is a biobank established for traditional Korean medicine (KM) that contains more than 20,000 clinical data collected since 2006. The aim of KDC is to verify and optimize the theory of Korean medicine, including SCM, as well as to develop preventive and novel therapies [20]. The dataset used in this study was constructed by recruiting and surveying 3891 unique patients who visited 13 traditional Korean medicine hospitals or 11 traditional Korean medicine clinics from November 2006 to July 2013. This multicenter registry dataset was compiled into the KDC of the Korea Institute of Oriental Medicine. All procedures were approved by the Korea Institute of Oriental Medicine (I-0910/02-001) and written informed consent for participation was obtained from each subject.

The surveyed data initially consisted of clinical information with various classes, including general information, characteristics, disease, SC types, and clinical laboratory measures. The SC-type diagnosis was conducted by an SCM expert, and subjects were included in this research only if their chief complaints were ameliorated after receiving SC treatment. It is noteworthy that this criterion can support the reliability of the diagnosis of SC types. To handle missing values, we omitted some features such as (i) features belonging to the clinical laboratory measures class and gynecology class, (ii) features belonging to symptoms with a low response rate, such as pain during bowel movement, in the stool feature group, and a certain amount of food in the diet feature group. Only patients who had all the remaining features were surveyed and included in the dataset. Finally, 1363 patients and 249 features (9 feature classes) were included in the subsequent analysis.

### 3.2. Entropy and Adjusted Mutual Information

Adjusted mutual information (MI) was used to calculate the degree of relevance between features or patients. MI is a measure of the amount of information that one random variable contains about another. The adjusted *MI* corrects for the bias in which the MI usually increases as the size of the vector increases, regardless of their actual association [21]. For the two feature vectors *U* and *V*, the adjusted *MI* is calculated as follows:

(1) $$\mathrm{Adjusted}\;\mathrm{MI}\left(U,V\right)=\frac{MI\left(U,V\right)-E\left\{MI\left(U,V\right)\right\}}{\max\left\{H\left(U\right),H\left(V\right)\right\}-E\left\{MI\left(U,V\right)\right\}}\;$$

where *MI* (*U*, *V*) is the mutual information between vectors *U* and *V* and can be calculated as follows:

(2) $$MI\left(U,V\right)=\overset{\left|U\right|}{\underset{i=1}{\sum }}\overset{\left|V\right|}{\underset{j=1}{\sum }}\frac{\left|U_{i}\cap^{\;}V_{j}\right|}{N}\;log\frac{N\;\left|U_{i}\cap^{\;}V_{j}\right|}{\left|U_{i}\right|\left|V_{j}\right|}\;$$

where *N* denotes the total number of dimensions of vectors *U* and *V* (i.e., the number of samples), and $\left|U_{i}\right|$ and $\left|V_{j}\right|$ denote the number of samples in clusters $U_{i}$ and $V_{j}$, respectively.

Entropy focused on the feature classes was used to measure the amount of information included in the SC type for the combination of feature classes. A random variable in entropy is defined as a feature class, and the probability by feature class is defined as the ratio of the sum of the adjusted MI between a target feature (i.e., SC type) and other features to the sum of the adjusted MI between target features and features belonging to the feature class. To assess how uncommon the derived value is, we calculated the entropy values for all features in the same manner and compared the relative ranks of the entropy values for the target features. This ranking was used to identify the combination of feature class information mainly included in the SC type. In other words, the combination of feature classes yielding a high relative ranking is the information mainly included in the feature of interest. AMI and entropy focused on the feature classes were calculated using scikit-learn 0.22.1.

### 3.3. Feature Network and Patient Network Construction

To construct the feature network, the adjusted MI between 249 features was calculated, and the distribution of the adjusted MI was visualized as a histogram and heatmap. To construct a binary network, the adjusted MIs were binarized to an adjacency matrix with only one (connected) or zero (unconnected) as elements by applying a threshold of 0.01 (Figure S1). This binarization process makes the networks tractable for the subsequent network-analysis approach. The feature network was represented as 249 nodes and 3259 edges, in which the nodes were clinical features and the edges between nodes were computed using the adjusted MI between features.

To construct the patient network, the adjusted MI between 503 male and 860 female patients was calculated using the 42 features connected with an SC-type feature in the feature networks. Male and female networks were constructed separately to reflect the heterogeneity of body shape and other characteristics. As the network separation value could only be calculated in a fully connected network, the largest MI that connected all nodes was used as the initial edge threshold (0.288 for males and 0.322 for females). The edge density of the male network was adjusted to the female network so that the difference in network properties did not arise from the difference in edge density. Finally, patient networks were constructed, which consisted of 503 nodes and 9684 edges in the male network and 860 nodes and 28,334 edges in the female network. In the patient network, nodes were patients, and the edges between nodes indicated the adjusted MI between patients. The adjusted MI was calculated using scikit-learn 0.22.1, and the feature and patient networks were visualized using Cytoscape 3.6.0 [22,23].

### 3.4. Network Separation between Feature Classes and Constitutions

Network separation is used to calculate the degree of separation between feature classes or patient modules (i.e., SC types) [24,25]. For node sets *A* and *B*, the network separation $s_{AB}$ is given as

(3) $$s_{AB}\;\equiv \;\langle d_{AB}\rangle -\frac{\langle d_{AA}\rangle +\langle d_{BB}\rangle }{2}\;$$

where *A* and *B* denote a feature module belonging to the same feature class or a patient module belonging to the same SC type, $\langle d_{AA}\rangle$ and $\langle d_{BB}\rangle$ denote the mean shortest distance inside each module, and $\langle d_{AB}\rangle$ denotes the mean shortest distance between modules *A* and *B*. A positive separation value indicates that the two modules are distinguished at the network level, whereas a negative separation value indicates that the two modules overlap. Feature classes isolated from other nodes (i.e., cancer feature groups) were excluded from the network separation calculation.

### 3.5. Statistical Analysis

Statistical analysis was performed according to the variable type (i.e., continuous variable and discrete variable). For multiple comparison tests between continuous variables, Shapiro–Wilk test was first used to assess whether the data were normally distributed. Then, one-way analysis of variance (ANOVA) followed by Dunnett’s test was used for the dataset with normality to compare unpaired multiple sample groups. When the normality was rejected, Kruskal–Wallis ANOVA followed by Dunn’s post hoc test was applied. For multiple comparison tests between discrete variables, chi-square tests were applied to compare multiple sample groups. All statistical significance was set at *p* < 0.05. The above analyses were performed using with SciPy module 1.7.1.

## 4. Results

### 4.1. Characteristics of Subjects and Features

We obtained a dataset from the Korean Medicine Data Center at the Korea Institute of Oriental Medicine. By applying the selection criteria described in the Methods section, we retrieved a dataset that consisted of 1363 patients (503 males and 860 females). The distribution of the population according to SC type for men was TE, 191; SE, 129; SY, 172; TY, 11. The distribution of women was as follows: TE, 312; SE, 263; SY, 267; and TY, 14. The dataset also contains 249 features that are useful information for the clinical practice of KM, including SC type. These features were grouped into nine feature classes as follows: general information, characteristics, symptoms, cold/heat, pathological symptoms, disease, health, body measure, and SC type. A detailed description of the feature classes and features is presented in Table 2.

### 4.2. Feature Network Construction and Analysis

**Figure 2:**
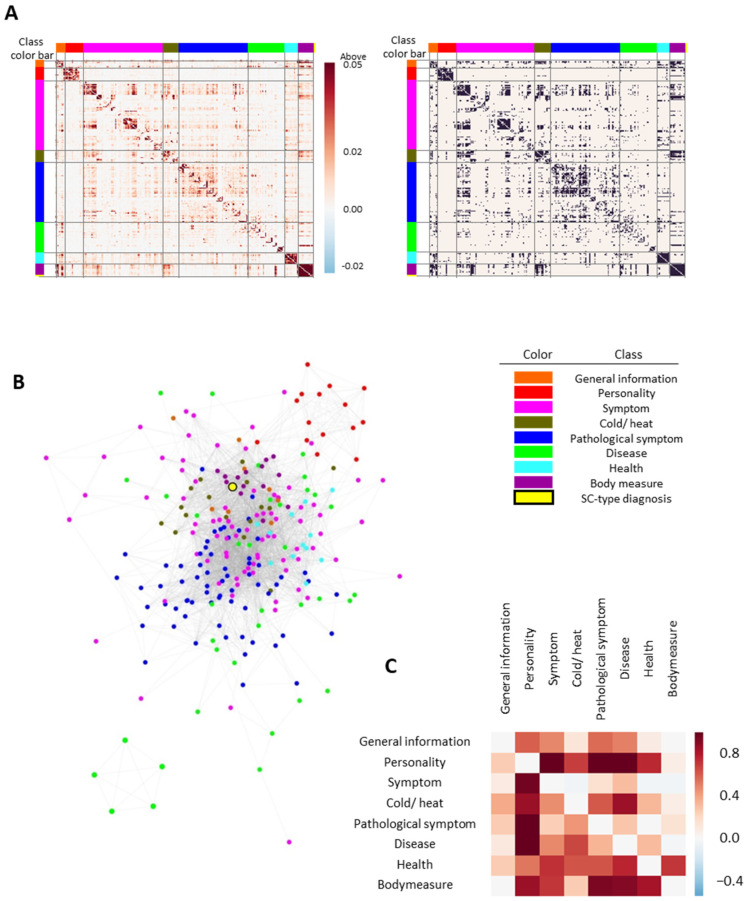
Construction and visualization of the feature network: (**A**) the association matrix between clinical features or SC types. Each feature in the heatmap is ordered according to its feature class. Data were visualized as raw adjusted mutual information (MI) values (**left**) or whether values exceeded the excess cutoff value (**right**); (**B**) visualized feature networks. Class color bar in the heatmap and the node color in the feature network indicate the feature class. Specifically, the yellow circle with black outline represents the SC-type diagnosis. The edges between nodes indicate that the MI value between features is greater than the cutoff value (0.01); (**C**) feature network separation between feature classes. Positive separation values indicate that two classes are well-distinguished at the network level, while negative values imply overlapping between two classes.

A feature network was constructed based on the adjusted MI to investigate the correlation between features or feature classes. The feature network consisted of 249 nodes and 3259 edges, where nodes refer to clinical information or SC types and edges denote associations between nodes. The network was closely connected between features belonging to the same classes. Densely connected features were also found between the features of different classes, such as general information–body measure, symptom–cold/heat, symptom–pathological symptom, cold/heat–body measure, and disease–body measure (Figure 2A,B). The close relationship between clinical and general information implies the potential of the multidimensional approach.

To describe the clinical characteristics of our network, we further investigated the associations between SC types and representative chronic diseases, such as hypertension, hyperlipidemia, diabetes, obesity, and cancer (Table 3). Generally, the adjusted MI of related features may seem slightly low; however, most related features show a statistically significant association with chronic diseases. However, related features associated with cancer showed a lower adjusted MI than those associated with other chronic diseases, and only five of them showed a significant correlation with SC types (*p* < 0.05). The consistency between the statistical analysis and adjusted MI values supports the reliability of the constructed network.

To quantitatively evaluate the degree of overlap in the network, network separation was calculated between features (Figure 2C). We found that most network separations between feature classes were positive values, indicating that features can be classified according to feature classes on the network. Specifically, the feature class pair containing the personality class exhibited the highest network separation value, such as personality symptoms and personality disease. This result indicates that personality classes can be distinguished from other feature classes. Nevertheless, some feature class pairs with the symptom class showed negative values (i.e., symptoms with cold/heat, health, or body measure), which suggests that the symptom class is correlated with other clinical information.

### 4.3. Features and Feature Classes Related to SC Type

We then tried to identify features and feature classes that were primarily related to the SC type. Statistical analysis was first used to identify features related to the SC type in the constructed network. The results showed that the SC type was mainly associated with features belonging to the body measure, cold/heat, and symptom classes (*p* < 0.0001 after Bonferroni correction). The feature group names, feature descriptions, and adjusted *p*-values of SC-associated features are summarized in Table 4.

**Figure 3:**
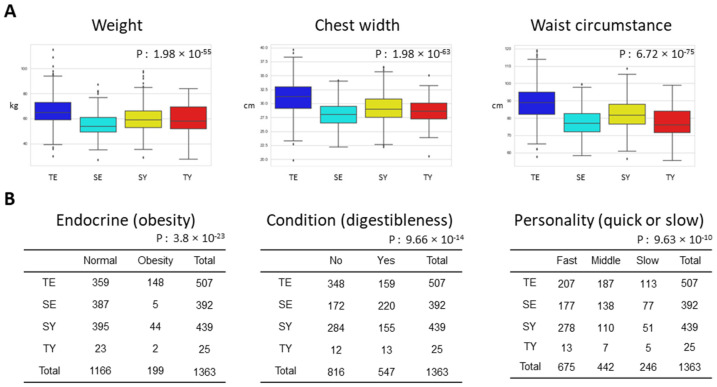
Representative examples of SC-associated features: (**A**) representative distributions of continuous variables according to SC type; (**B**) representative distributions of discrete variables according to SC types. The names of the feature are represented by the status of the feature class (feature name).

To describe the analysis results, representative example distributions of SC-associated features are presented. We found that the weight, chest width, and waist circumference showed that TE was higher and SE was lower than that of the other constitutions. The proportion of obesity was also significantly higher in TE, and SE was much lower than in other constitutions (Figure 3A). In addition, the proportion of condition_digest (rate of poor digestion when conditions are bad) was lower in TE, whereas it was much higher in SE than in other constitutions. The proportion of Character Fast slow was significantly different according to the constitution; TE had a higher proportion of middle and slow (36.9%, 22.2%), and SY had a higher proportion of fast than other constitutions (63.3%, 52.0%, 45.2%, and 40.8% for SY, TY, SE, and TE, respectively; Figure 3B).

**Figure 4:**
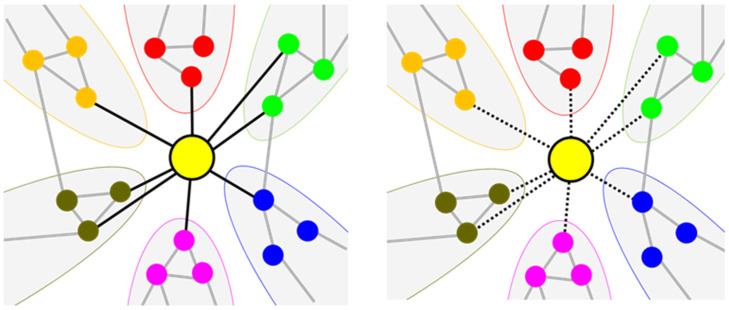
Entropy modeling for SC type. The left figure shows the possible state that the SC type is connected to the nodes of various feature classes, which indicates that the SC type includes information about various classes. On the other hand, the right figure indicates another possible state that the SC type has no connection, which indicates that the SC type contains limited information.

To identify related feature classes, we calculated the entropy ranking of the SC type, focusing on feature class combinations. We expected that by identifying combinations with high relative rankings, we could identify the feature classes mainly included in the SC type (Figure 4). The hypotheses and rationale for this metric are summarized in the Methods section. We first evaluated whether the SC type contained information on all feature classes. When all feature classes were used, we found that the ranking of the SC type was not high (80/249), indicating the possibility that the SC type does not evenly include information on all feature classes. Then, we attempted to identify which class information the SC type mainly contained by calculating the relative ranking using all possible combinations of feature classes (Table 5). We found that the relative ranking of SC types was highest in the combination of personality and cold/heat classes. In addition, these two features appeared the most in the top-10 combinations with a high relative rank of SC type (*n* = 8 and 6 for personality and cold/heat, respectively). These results suggest that the SC type primarily incorporates this information.

### 4.4. Patient Network Construction and Analysis

**Figure 5:**
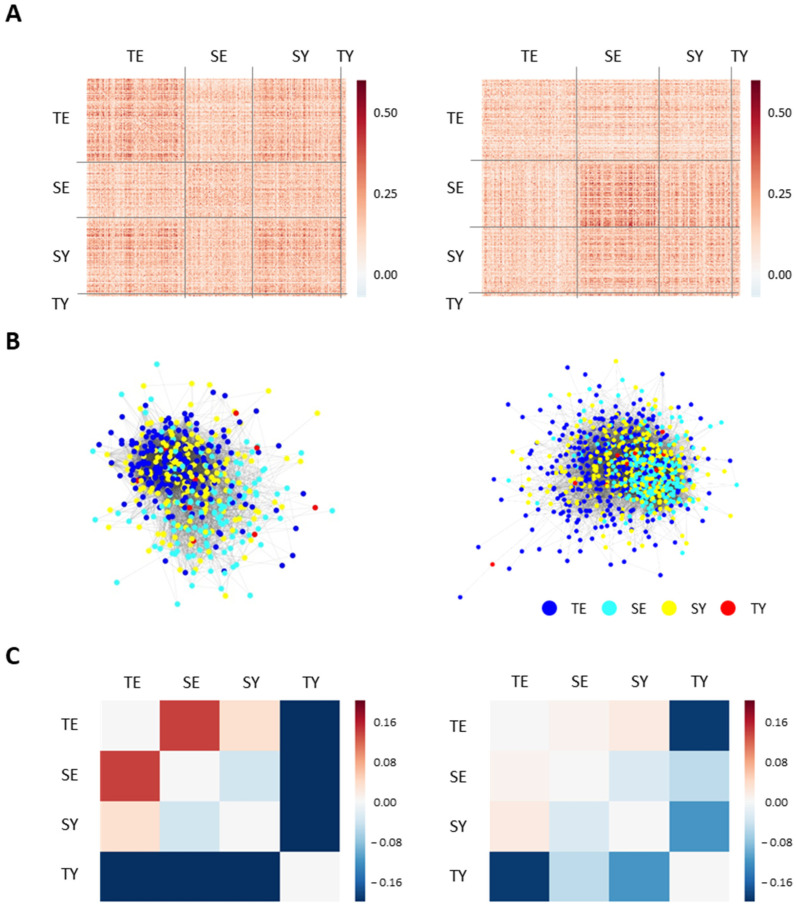
Patient network for men (**left column**) and women (**right column**): (**A**) distribution of adjusted MI value between patients; (**B**) the visualized patient networks. The nodes in the patient network represent patients, and the colors of each node indicate SC type. The edges of the network adjusted MI scores between patients; (**C**) the patient network separation between SC types. Positive separation values indicate that two SC types are well-distinguished at the network level, while negative values imply overlapping between two SC types.

The patient network was constructed based on the MI between subject pairs. The male and female patient networks were constructed separately to reflect the heterogeneity between genders. The male network consisted of 503 nodes and 9684 edges, and the female network was composed of 860 nodes and 28,334 edges (Figure 5A,B). In the constructed network, male TE, SY, and female SE were closely connected to patients with the same SC types. In addition, the TE–SY pair in the male network and the SE–SY pair in the female network were closely connected. The close connection between the same SC types or other SC types indicates that a specific SC-type pair has higher similarities than other SC types.

Network separation between SC pairs was calculated to quantitatively assess topological overlap between SC types (Figure 5C). The separation values of TE–SE and TE-SY were positive, while the values of SE–SY and all TY-related pairs, TY-TE, TY-SE, and TY-SY, were negative in both sexes. The separation value between the TE–SE pair in the female network was much lower than that of the male, and the overall deviation of separation values in the female network was lower than that of the male network. This result indicates that there is a unique pattern of topological separation according to SC type and sex. It is noteworthy that we adjusted the edge densities equally between these networks, indicating that our finding did not arise from differences in edge densities. Taken together, we identified the relationships and separations between SC types through network construction and analysis.

## 5. Discussion

SCM is a unique concept of KM that can provide valuable insights into personalized healthcare and disease treatment [3]. Despite its usefulness, current approaches have been limited to identifying the association between SC type and a set of predefined diseases or symptoms. In this study, we comprehensively elucidated the complex relationships between SC types and clinical information by combining network analysis and information entropy. The feature network revealed a comprehensive relationship and closeness between clinical information and SC type. We identified 42 SC type-related features majorly included in the personality, symptoms, and body measure classes. The results of the entropy model suggested that the information on the SC type was mainly concentrated on pathological symptoms, diseases, and cold-heat classes. The patient network results suggest the closeness of the SC types by measuring the degree of topology separation.

To the best of our knowledge, our study is the first to systematically identify features and feature classes related to SC type in a hypothesis-free manner. Firstly, we comprehensively analyzed the topological characteristics of feature and patient networks, which provide a deeper understanding between SC types and other clinical information. Our data-driven approach proposes that the SC type can be a practically useful concept to accelerate stratified healthcare and personalized medicine. Additionally, we also suggest that applying a network analytical approach to high-dimensional clinical datasets could provide deeper insights into the complex relationships between clinical information.

The entropy ranking results suggested that personality and cold/heat were the major information contained in the SC type. Interestingly, the discovered feature classes related to the SC type provide valuable information empirically used in the disease diagnosis and selection of herbal medicines. According to the SCM, personality is a major factor influencing the movement of the *qi* of internal organs, and it contributes to the formation of physiological and pathological mechanisms that determine the function and structure of the human body [26,27,28]. Cold/heat information is a fundamental concept used as an indicator to understand the pathophysiological status of patients and to diagnose drug or acupuncture prescriptions in KM and SCM [29,30,31]. This result is in line with the core idea of SCM, which treats people by considering the state of their body and mind together.

The feature network found densely connected modules between features, such as general information-body measure, symptom-cold/heat, and symptom-pathological symptom classes (Figure 2). These modules not only represent close relations between each class pair but also suggest a potential relationship between feature pairs belonging to each class. Some studies have investigated the relevance between clinical information and diseases that can support the relationship between feature class pairs presented in our network. For example, one study revealed an association between cold hands and feet and functional dyspepsia [32], which is associated with our results of the densely connected module between the cold/heat class and the symptom class. We also measured the degree of overlap between feature classes using network separation. Three of the four class pairs with negative network separation values were associated with the symptom class, suggesting that this class is correlated with other clinical information. In addition, the statistical analysis focused on chronic diseases and provided relevant clinical information. We found previous research supporting the discovery of related factors. For example, our network showed that age, marriage features, and features in the body measure class were closely related to most chronic disease features, which have been indicated as demographic and general health factors associated with chronic diseases [33,34]. In addition, the associations between diabetes and some cardiovascular diseases (i.e., hypertension and hyperlipidemia) are consistent with the results of representative cohort studies [35]. Overall, comparisons with previous studies and the constructed network support the reliability of feature networks.

The results of the patient network systematically revealed the degree of topological separation between SC types. The TY type was not topologically separated from all other SC types, which could be due to the lower numbers compared to the other SC types or the lower similarity to the TY type. We found that two TE-related pairs, TE–SE and TE-SY, were sparsely connected in the patient network and showed positive network separation values. This indicates that the TE constitution is topologically separable from the SE and SY types. In addition, the SE–SY pair showed a dense connection in the patient network and had a negative network separation value between them. The association between our network-level findings and the prediction results of previous machine learning can be interpreted as the following two possibilities. One possibility for this result is that SC-type pairs isolated in the network are difficult to distinguish in actual diagnosis. In other words, topologically separable pairs of SC types are also easily distinguished in clinical practice. Previous studies on predicting SC types reported that prediction performance was highest in the TE type and lowest in the SY type [18,36,37], which is consistent with our results. Another possibility is that our results may have limited applicability to the ease of diagnosis. Actually, the previous machine learning approach suggested that the key diagnostic symptoms or indicators for constitutional diagnosis differ by the constitution, such as body type in the TE–SE pair and personality in the SE–SY pair [18]. This difference may be because our analysis is based on major features related to SC type.

Potential limitations may be derived from our study. First, the degree of topological separation between the constitution may have been perturbed by the distribution of SC types belonging to the dataset. Second, our dataset consists of patients who have visited the hospital and does not include healthy people. Therefore, our future direction is to collect and analyze more data samples, including healthy individuals, and compare the results with this study. Additionally, it will be interesting to compare our research results with the expert knowledge of SCM.

## Figures and Tables

**Table 1:** Comparison with previous studies on clinical data analysis on Sasang constitution.

	Domain	Approach Type	Task Type
This work	General	Data-driven	Exploratory analysis
Jang et al. (2015) [10]	Hypertension	Hypothesis-driven	Risk factor investigation
Baek et al. (2014) [11]	Obesity	Hypothesis-driven	Prevalence investigation
Lee et al. (2009) [12]	Diabetes mellitus	Hypothesis-driven	Risk factor investigation
Kim et al. (2022) [13]	Depressive symptoms	Hypothesis-driven	Risk factor investigation
Jeong et al. (2020) [14]	General	Hypothesis-driven	Dietary behaviors evaluation
Lee et al. (2020) [15]	General	Hypothesis-driven	Urinary function evaluation
SCAT [16]	General	Data-driven	SC type Diagnosis
K-prism [17]	General	Data-driven	SC type Diagnosis
Park et al. (2020) [18]	General	Data-driven	SC type Diagnosis
Kim et al. (2022) [19]	General	Data-driven	SC type Diagnosis

**Table 2:** A description of the feature classes and feature groups in the dataset.

Feature Class	Feature Group	Number of Features	Description of Feature Group
General information	General information	8	Gender, height, weight, occupation, education, Sibling relationship, marriage
Personality	Personality	15	Sasang Personality Questionnaire
Symptoms	Diet	4	General information about diet
	Digest	13	Abnormal digestive symptoms
	Sweat	18	Response and area of sweating
	Stool	21	Frequency and character of stools, and accompanying symptoms
	Urine	13	Frequency and character of urine, and accompanying symptoms
	Water	3	General information about water drink
	Sleep	8	Sleep time, accompanying symptoms, and sleep quality
Cold/heat	Cold/heat	16	Symptom questionnaire for heat, cold pattern identification
Pathological symptoms	Head	10	Pain or discomfort symptoms in head
	Body	5	Pain or discomfort symptoms in body
	Eye	6	Pain or discomfort symptoms in eye
	Mouth	7	Discomfort symptoms in mouth
	Chest	6	Pain or discomfort symptoms in chest
	Skin	3	Discomfort symptoms in skin (i.e., Dryness, itching or others)
	Fatigue	6	Time and severity of fatigue
	Cold	11	Discomfort symptoms (i.e., fever, headache, dizziness, etc.) when you have a cold
	Bad condition	6	Discomfort symptoms (i.e., sweat, digestion, stool, urine, etc.) when conditions are bad
	Other symptoms	7	Other uncomfortable symptoms (e.g., edema, forgetfulness)
Disease	Hypertension	1	Whether to diagnose hypertension
	Diabetes	1	Whether to diagnose diabetes
	Hyperlipidemia	1	Whether to diagnose hyperlipidemia
	Osteoporosis	1	Whether to diagnose osteoporosis
	Muscular skeletal disease	4	Whether to diagnose herniated intervertebral disc, degenerative arthritis, rheumatoid arthritis
	Cardiovascular disease	4	Whether to diagnose anemia, angina pectoris, stroke
	Gastrointestinal disease	6	Whether to diagnose chronic gastritis, gastroduodenal ulcer disease, fatty liver, hepatitis
	Pulmonary disease	6	Whether to diagnose pneumonia, asthma, chronic sinusitis, tuberculosis
	Endocrine disease	4	Whether to diagnose Hypothyroidism, hyperthyroidism, obesity
	Cancer	6	Location and type of cancer
	Other	2	Operation history, other diseases
Health	Health	12	12-Item Short Form Survey
Body measure	Body measure	14	Width of axillary, chest, rib, waist, pelvic, and circumstance of forehead, neck, axillary, chest, rib, waist, pelvic, hip, and angle of belly
Constitution Diagnosis	Constitution Diagnosis	1	Constitution diagnosis by doctor of Korean medicine

**Table 3:** Features related to representative chronic diseases.

Chronic Disease	Related Feature	Class of Related Feature	Adjusted MI	Adjusted *p*-Value ^#^
Hypertension	Age	General information	0.084	2.01 × 10^−57^
	Stroke	Disease	0.083	9.75 × 10^−32^
	Rib circumference	Body measure	0.057	5.94 × 10^−38^
	Chest circumference	Body measure	0.050	9.76 × 10^−31^
	Marriage	General information	0.050	7.97 × 10^−18^
	Hyperlipidemia	Disease	0.050	1.72 × 10^−18^
Hyperlipidemia	Hypertension	Disease	0.050	1.72 × 10^−18^
	Fatty liver	Disease	0.031	1.17 × 10^−9^
	Age	General information	0.030	2.37 × 10^−12^
	Chest circumference	Body measure	0.026	7.33 × 10^−13^
	Rib circumference	Body measure	0.023	5.32 × 10^−14^
	Diabetes	Disease	0.023	5.59 × 10^−7^
Diabetes	Stroke	Disease	0.074	9.93 × 10^−23^
	Foamy urine	Symptom	0.042	2.98 × 10^−12^
	Age	General information	0.040	3.94 × 10^−27^
	Hypertension	Disease	0.038	2.72 × 10^−14^
	Night urine frequency	Symptom	0.027	5.52 × 10^−19^
	Marriage	General information	0.022	4.83 × 10^−7^
Obesity	Chest circumference	Body measure	0.072	5.87 × 10^−48^
	Fatty liver	Disease	0.068	1.39 × 10^−22^
	Waist circumference	Body measure	0.054	3.78 × 10^−40^
	Waist width	Body measure	0.054	9.69 × 10^−42^
	Pelvic circumference	Body measure	0.052	3.22 × 10^−43^
	SC diagnosis	SC type	0.052	9.58 × 10^−31^
Cancer	Operation history	Disease	0.064	3.71 × 10^−22^
	Marriage	General information	0.012	0.22
	Gender	General information	0.011	9.06 × 10^−3^
	Other symptoms *	Symptom	0.009	0.01
	Dizziness	Symptom	0.009	0.02
	Cold/heat in abdomen	Cold-heat	0.008	2.80 × 10^−3^

* Other symptoms: swelling, forgetfulness, dizziness, powerlessness in the leg, swelling, heat in joints, or other symptoms. ^#^ Bonferroni correction was applied for multiple comparison tests.

**Table 4:** Description of features related to Sasang Constitution.

Feature Group Name	Feature Description	Adjusted *p*-Value #
Weight	Weight	1.98 × 10^−55^
Personality	Do you consider yourself feminine or masculine?	1.04 × 10^−6^
	Do you consider yourself a delicate or tough personality?	6.81 × 10^−7^
	Do you consider yourself relatively lethargic or energetic?	1.59 × 10^−9^
	Do you consider yourself introverted or extroverted?	1.88 × 10^−6^
	Do you consider yourself passive or proactive?	1.37 × 10^−8^
	Do you consider yourself quick-tempered or slow?	9.63 × 10^−10^
Sweat	How much do you sweat when you are hot?	6.52 × 10^−11^
	How much do you sweat when you exercise?	8.22 × 10^−13^
	How much do you sweat during your daily life?	1.38 × 10^−11^
	How often do you usually sweat?	1.96 × 10^−14^
Digest	How about your usual taste?	5.82 × 10^−7^
	Do you usually have abdominal pain?	7.66 × 10^−12^
Water	How much water do you usually drink?	4.86 × 10^−10^
	What is the temperature of the usual drinking water?	8.53 × 10^−6^
Cold/heat	Which do you not prefer, cold or hot?	2.83 × 10^−10^
	Are your hands cold or warm?	8.34 × 10^−10^
	Are your foots cold or warm?	5.33 × 10^−11^
	Warm energy or warm temperatures are good.	1.56 × 10^−8^
	My limbs are cold	1.42 × 10^−8^
	My face looks pale	5.83 × 10^−10^
	Cool temperatures and stimulation are good.	2.22 × 10^−13^
	My body feels a fever and a hot symptom.	5.27 × 10^−11^
Condition	When the body condition is bad, there is a problem in digestion	9.66 × 10^−14^
Hypertension	Have you ever been diagnosed with hypertension?	9.55 × 10^−10^
Endocrine disease	Have you ever been diagnosed with an endocrine disorder?	1.92 × 10^−32^
	Have you ever been diagnosed with obesity?	3.8 × 10^−23^
Gastrointestinal disease	Have you ever been diagnosed with a fatty liver?	4.59 × 10^−10^
Body measure	Axillary width	5.26 × 10^−45^
	Chest width	1.98 × 10^−63^
	Rib width	7.8 × 10^−47^
	Waist width	4.68 × 10^−67^
	Pelvic width	3.47 × 10^−20^
	Forehead circumference	1.5 × 10^−14^
	Neck circumference	7.69 × 10^−38^
	Axillary circumference	3.32 × 10^−59^
	Chest circumference	2.04 × 10^−78^
	Rib circumference	1.18 × 10^−67^
	Waist circumference	6.72 × 10^−75^
	Pelvic circumference	4.73 × 10^−67^
	Hip circumference	2 × 10^−53^
	Belly angle	1.75 × 10^−59^

# Bonferroni correction was applied for multiple comparison tests.

**Table 5:** Feature class combination related to SC type according to relative entropy ranking.

Combinations of Feature Class	Relative Rank (Percentile)
Personality, cold/heat	13 (5.2)
Personality, symptom, cold/heat	24 (9.6)
Pathological symptoms, disease	28 (11.2)
Personality, body measure, cold/heat	28 (11.2)
Personality, health, cold/heat	36 (14.5)
Health, symptom	40 (16.1)
Personality, health, symptom, cold/heat	53 (21.3)
Personality, body measure	55 (22.1)
Personality, health, symptom	56 (22.5)
Personality, pathological symptoms, cold/heat	56 (22.5)

## Data Availability

The data supporting the findings of this study are available from the Korea medicine Data Center (KDC). Data can be made available from the authors upon reasonable request and with permission of the KDC and the Institutional Data Access/Ethics Committee of the Korea Institute of Oriental Medicine. Requests for access to these data can be made at the KDC website (kdc.kiom.re.kr). Source codes used in this study are available at: https://github.com/wonyung-lee/SC_information_entropy (accessed on 3 November 2022).

## Supplementary Materials

The following supporting information can be downloaded at: https://www.mdpi.com/article/10.3390/healthcare10112248/s1. Figure S1: Adjusted mutual information distribution between feature pairs.
