# Supplementary figures and images for: Exploratory Analysis of the Sasang Constitution by Combining Network Analysis and Information Entropy
Source: Healthcare (Basel). 2022 Nov 10;10(11):2248. doi: 10.3390/healthcare10112248 (PMC9690606; doi:10.3390/healthcare10112248)

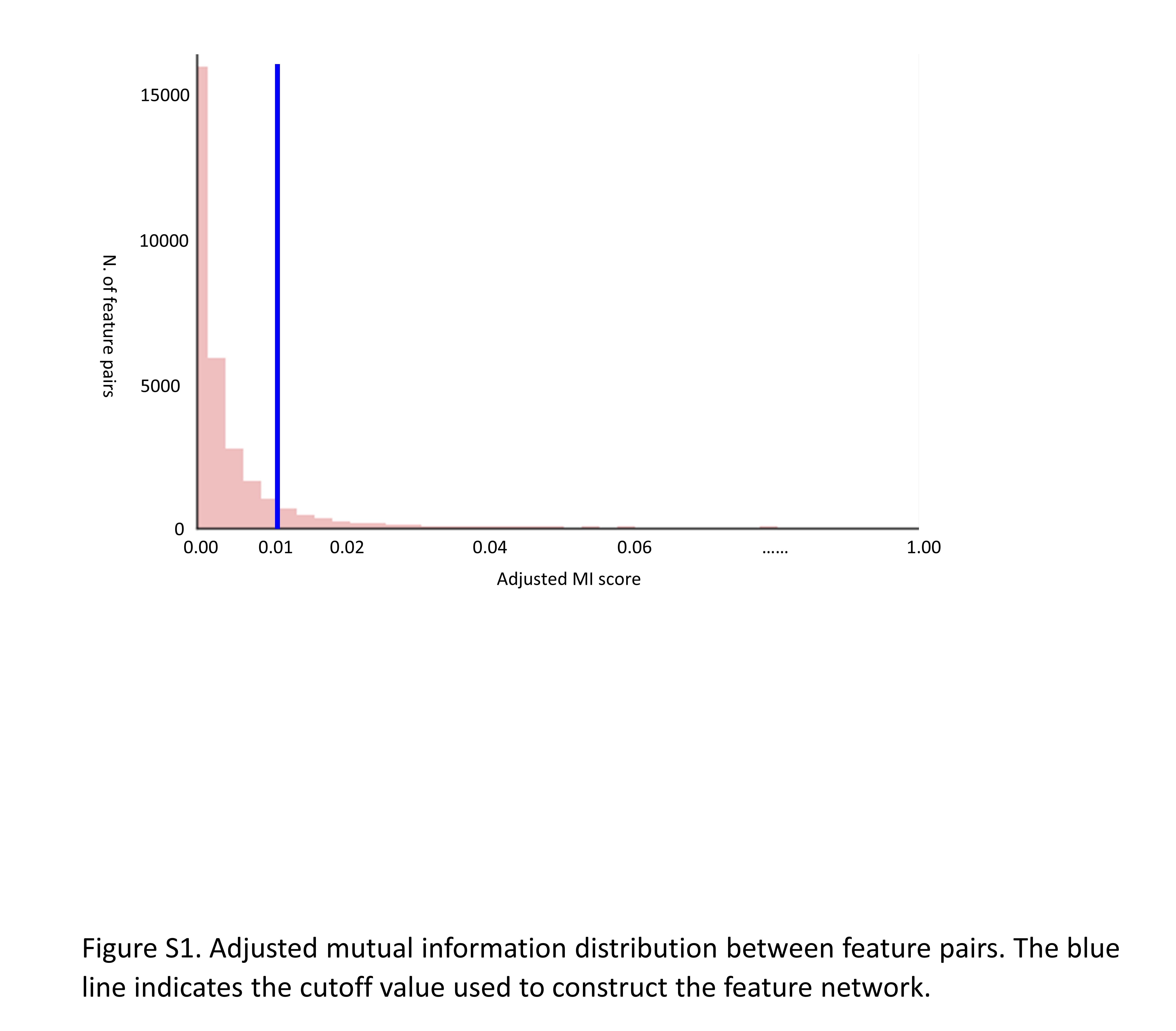

Supplement: Supplementary file 1 [file healthcare-10-02248-s001.zip › healthcare-1976850-supplementary.jpg]
